# Preferential attachment in the evolution of metabolic networks

**DOI:** 10.1186/1471-2164-6-159

**Published:** 2005-11-10

**Authors:** Sara Light, Per Kraulis, Arne Elofsson

**Affiliations:** 1 Stockholm Bioinformatics Center, Department of Biochemistry and Biophyhsics, Albanova University Center, Stockholm University, Stockholm SE-10691, Sweden

## Abstract

**Background:**

Many biological networks show some characteristics of scale-free networks. Scale-free networks can evolve through preferential attachment where new nodes are preferentially attached to well connected nodes. In networks which have evolved through preferential attachment older nodes should have a higher average connectivity than younger nodes. Here we have investigated preferential attachment in the context of metabolic networks.

**Results:**

The connectivities of the enzymes in the metabolic network of *Escherichia coli *were determined and representatives for these enzymes were located in 11 eukaryotes, 17 archaea and 46 bacteria. *E. coli *enzymes which have representatives in eukaryotes have a higher average connectivity while enzymes which are represented only in the prokaryotes, and especially the enzymes only present in βγ-proteobacteria, have lower connectivities than expected by chance. Interestingly, the enzymes which have been proposed as candidates for horizontal gene transfer have a higher average connectivity than the other enzymes. Furthermore, It was found that new edges are added to the highly connected enzymes at a faster rate than to enzymes with low connectivities which is consistent with preferential attachment.

**Conclusion:**

Here, we have found indications of preferential attachment in the metabolic network of *E. coli*. A possible biological explanation for preferential attachment growth of metabolic networks is that novel enzymes created through gene duplication maintain some of the compounds involved in the original reaction, throughout its future evolution. In addition, we found that enzymes which are candidates for horizontal gene transfer have a higher average connectivity than other enzymes. This indicates that while new enzymes are attached preferentially to highly connected enzymes, these highly connected enzymes have sometimes been introduced into the *E. coli *genome by horizontal gene transfer. We speculate that *E. coli *has adjusted its metabolic network to a changing environment by replacing the relatively central enzymes for better adapted orthologs from other prokaryotic species.

## Background

Recent studies indicate that metabolic networks evolve at the local level through patchwork evolution and retrograde evolution [1-3]. Patchwork evolution, which is likely to be more important, occurs when an enzyme evolves from a broad spectrum enzyme to an enzyme with a highly specialized activity [4]. Retrograde evolution is a process where the depletion of a substrate from the environment leads to the evolution of an enzyme which can accept a new substrate and catalyze the production of the depleted substance [5].

**Figure 1:**
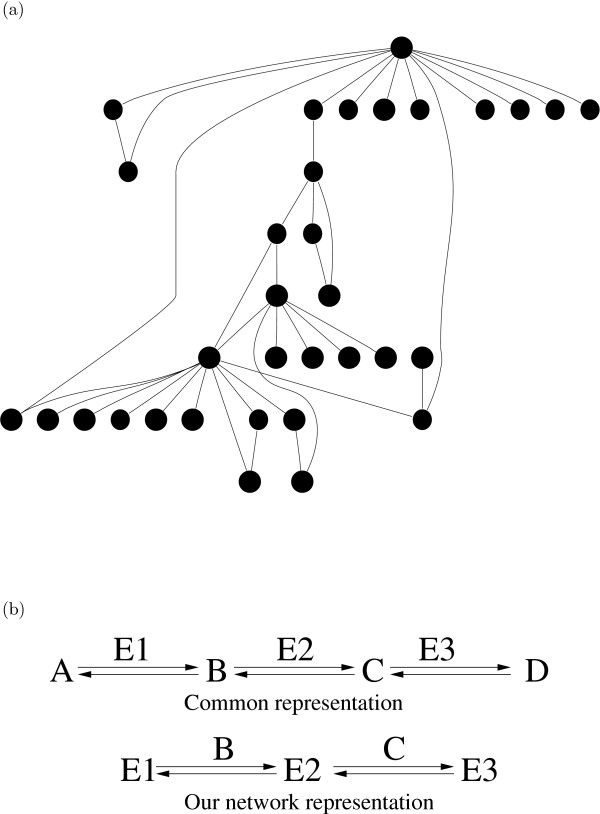
**Properties of scale-free networks and network representation**. a) The figure shows a network where most nodes have very low connectivities (k = 1) but two nodes have connectivities which are far higher than the connectivity of most nodes in the network (k = 12). Scale-free networks, among other networks, have this general property. b) The upper part of the figure shows a common network representation where the substrates and products of the reactions represent the nodes in the network and the enzymes represent the edges. Our network representation is shown in the lower part of the figure where the enzymes represent the nodes in the network and the substrates and products represent the edges (reaction graph).

Metabolic networks and other complex networks such as the film actor collaboration network, the world wide web, protein domain networks and protein-protein interaction networks are small-world networks with some properties which are consistent with scale-free networks [6-8]. The small-worldness of the metabolic network of *E. coli *has recently been contested for an alternative network representation where carbon atomic traces in metabolic reactions were used [9]. A small-world network is characterized by 1) short path lengths between any two nodes in the network and 2) a high clustering coefficient, which means that the neighbors of a certain node of the network are often connected to each other thereby forming clusters. A scale-free network, in this context, has a power-law connectivity (degree) distribution, i.e. there are many nodes which have very low connectivities and a handful of nodes with much higher connectivities (hubs), see Figure 1a. Scale-free networks are robust networks in the sense that they often remain intact when a large fraction of randomly chosen nodes is eliminated from the network [10]. However, if a small fraction of the hubs of the network is eliminated the network is likely to become fragmented into several components. It has been suggested that the scale-free character of biological networks has evolved through natural selection for the advantage of robustness and error-tolerance that the scale-free network topology confers to the organism [6]. A study by Gleiss *et al *showed that chemical reaction networks, which clearly have not been subjected to natural selection, also show scale-free characteristics thereby showing that scale-free networks can arise without natural selection and may be a general feature of chemical reaction networks [11].

Networks with scale-free properties have been shown to evolve when two simple rules are applied: 1) The network grows by the addition of new nodes. 2) Preferential attachment: New nodes are more likely to become connected to well connected nodes in the network [12]. While preferential attachment is often at the root of scale-freeness, a network with an power-law degree distribution might be produced through other mechanisms. Preferential attachment in the context of genetic networks may take place partly through gene duplication [13,14]. In agreement with preferential attachment Eisenberg and Levanon [15] showed that the proteins which have homologs in all 3 domains of life, which are likely to be of ancient origin, have higher connectivities in the protein-protein interaction network of *S. cerevisiae*. In contrast, Kunin *et al *[16] recently showed that the most highly connected proteins date to after the evolution of primordial eukaryotes but before the radiation of eukaryotes to Plants, Metazoa and Protista. Here, we investigate the evidence for preferential attachment and the role of horizontal gene transfer in the metabolic network evolution of *E. coli*.

## Results

### Connectivity and phylogenetic group

If preferential attachment is an important mechanism in the evolution of metabolic networks older enzymes should have a higher average connectivity (k) than younger enzymes. In order to investigate this prediction we extracted the enzymes and the reactions in *E. coli *from the EcoCyc [17] and KEGG [18] databases. The network representation of the metabolic network of *E. coli *was constructed using EcoCyc, see methods. The nodes in our graph represent the enzymes (complete EC numbers) catalyzing the reactions and the edges represent one or more compounds involved in the reactions. There is an edge from enzyme El to enzyme E2 if El catalyzes a reaction where compound A is produced and then E2 uses A as substrate. There can be at most one edge in each direction between the nodes in the graph. The connectivity of a node is defined as the number of edges connecting the node to other nodes in the network.

**Figure 2:**
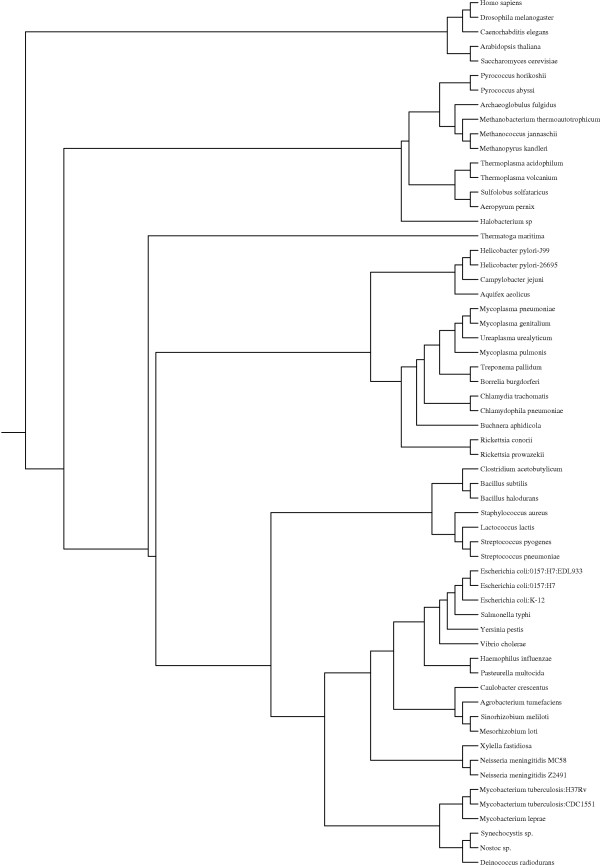
**Schematic representation of the phylogenetic tree for most of the organisms used in this analysis**. The tree was redrawn from Gough *et al *[35] using Drawgram from the Phylip package . Eukaryotes: *Homo sapiens*, *Caenorhabditis elegans*, *Drosophila melanogaster*, *Saccharomyces cerevisiae*, *Arabidopsis thaliana*, archaea: *Aeropyrum pernix*, *Sulfolobus solfataricus*, *Thermoplasma volcanium*, *Thermoplasma acidophilum*, *Methanopyrus kandleri*, *Methanococcus jannaschii*, *Methanobacterium thermoautotrophicum*, *Archaeoglobus fulgidus*, *Pyrococcus abyssi*, *Pyrococcus horikoshii*, *Halobacterium sp*. and bacteria: *Campylobacter jejuni*; Thermatogales: *Thermotoga maritima*; Parasitic proteobacteria: *Rickettsia conorii*, *Rickettsia prowazekii, Buchnera aphidicola*; Chlamydiae: *Chlamydophila pneumoniae*, *Chlamydia trachomatis*; Spirochetes: *Borrelia burgdorferi*, *Treponema pallidum*; Mycoplasmas: *Mycoplasma genitalium*, *Mycoplasma pneumoniae*, *Ureaplasma urealyticum*, *Mycoplasma pulmonis*; Bacillus/Clostridium-group: *Caulobacter crescentus*, *Staphylococcus aureus*, *Bacillus halodurans*, *Bacillus subtilis*, *Lactococcus lactis*, *Streptococcus pneumoniae*, *Streptococcus pyogenes*, *Clostridium acetobutylicum*; Cyanobacteria: *Nostoc sp*., *Synechocystis sp*.; Thermus/Deinococcus-group: *Deinococcus radiodurans*; Actinobacteria: *Mycobacterium tuberculosis CDC1551*, *Mycobacterium tuberculosis H37Rv*, *Mycobacterium leprae*; free-living α-proteobacteria: *Agrobacterium tumefaciens*, *Mesorhizobium loti*, *Sinorhizobium meliloti*; ε-proteobacteria: *Helicobacter pylori-J99*, *Helicobacter pylori-26695*; Aquificales: *Aquifex aeolicus *and βγ-proteobacteria: *Escherichia coli:0157:H7*, *Escherichia coli:0157:H7:EDL933*, *Escherichia coli:k-12*, *Salmonella typhimurium*, *Yersinia pestis*, *Vibrio cholerae*, *Neisseria meningitidis 72491*, *Neisseria meningitidis MC58*, *Xylella fastidiosa*, *Pseudomonas aeruginosa*, *Ralstonia solanacearum*, *Pasteurella multocida*, *Haemophilus influenzae*. Furthermore, 6 additional eukaryotes (*Schizosaccharomyces pombe*, *Plasmodium falciparum*, *Encephalitozoon cuniculi*, *Mus musculus*, *Rattus norvegicus*, *Danio rerio*) and 6 additional archaea (*Methanosarcina mazei*, *Methanosarcina acetivorans*, *Nanoarchaeum equitans*, *Pyrobaculum aerophilum*, *Pyrococcus furiosus*, *Sulfolobus tokodaii*) were used in the analysis.

Enzymes (complete EC numbers) were collected from KEGG orthology [18] and were found in 163 different organisms (11 eukaryotes, 17 archaea and 135 bacteria). Among these the *E. coli *enzymes with representatives in 74 organisms (11 eukaryotes, 17 archaea and 46 bacteria) of reasonably well understood phylogenies were extracted for further studies, see Figure 2. The enzymes were divided into five age groups, see Table 1. The enzymes in group 1 are likely to be among the oldest since they have representatives in eukaryotes, archaea and bacteria. Group 2 contains the enzymes with representatives in eukaryotes and bacteria but not in archaea while group 3 contains the enzymes with representatives in archaea and bacteria but not in eukaryotes. Group 4 contains *E. coli *enzymes without representatives in eukaryotes or archaea but with representatives in bacteria other than γβ-proteobacteria and group 5 contains enzymes with representatives in γβ-proteobacteria only. Enzymes belonging to group 1–3 are probably ancient enzymes since they exist in at least two domains of life. Enzymes which are found in group 5 are only found in bacteria which are comparatively close relatives of *E. coli*, in the γβ-proteobacteria group, see Figure 2, which indicates that they are relatively recent additions to the metabolic repertory of *E. coli*. It is possible that some of the enzymes in groups 1–3 could have evolved relatively recently and subsequently been horizontally transferred to the other domains of life but the evidence of horizontal gene transfer between organisms belonging to different domains of life is not abundant. Horizontal gene transfer between bacterial species is believed to be more common [19] and therefore there may be enzymes in group 4 which have been transferred from γβ-proteobacteria. Gene loss, which is estimated to be three times more common than horizontal gene transfer in prokaryotes [20], is probably a more important source of error in this study since genes which serve an important function in bacteria but not in archaea or eukaryotes may have been lost in the archaeal and eukaryotic lineages and as a consequence groups 4 and 5 may not exclusively contain relatively recently evolved enzymes.

**Table 1:** Description of the phylogenetic groups 1–5 and the number of *E. coli *enzymes in each group. For instance, an *E. coli *enzyme which has at least one representative in one or more eukaryotes but not in archaea is a group 2 enzyme. The fourth column contains the number of enzymes which are proposed examples of horizontal gene transfer. The phylogenetic classification is based on the phylogenetic tree in Figure 2.

GROUP	ORGANISMS	NO. ENZYMES	NO. HGT ENZYMES
1	*E. coli*, eukaryotes and archaea	262	45
2	*E. coli*, eukaryotes but not archaea	71	14
3	*E. coli *and archaea	50	8
4	*E. coli *and bacteria other than βγ-proteobacteria	75	14
5	βγ-proteobacteria	28	4

**Figure 3:**
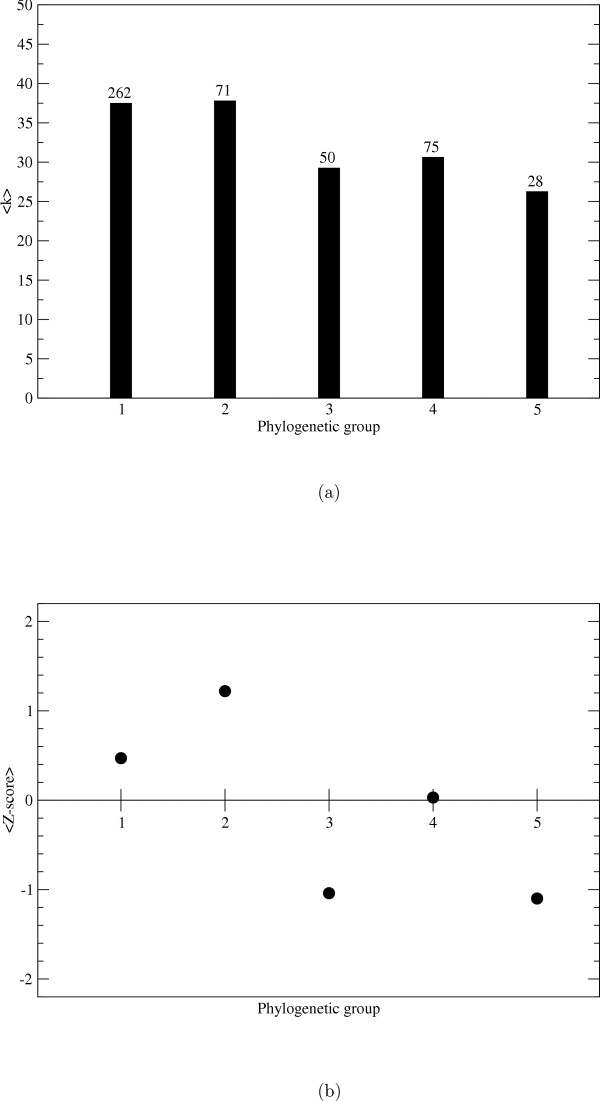
**The connectivity of enzymes belonging to different phylogenetic groups**. a) Average connectivity for enzymes in phylogenetic groups 1–5 in the metabolic network where the 15 most promiscuous compounds have been removed. b) The average Z-score (between 15–20 compounds removed from the network) is plotted for the phylogenetic groups 1–5.

The average connectivities for the *E. coli *enzymes with representatives in the five groups were calculated, see Figure 3a. The enzymes which are represented in all 3 domains of life (group 1) have the highest average connectivity together with the enzymes which occur in bacteria and eukaryotes (group 2) while the enzymes that occur only in γβ-proteobacteria (group 5) have the lowest connectivities. The average connectivity was 40% higher for group 1 than for group 5 enzymes. In order to estimate the significance of the results 100 000 randomized networks were generated through shuffling the group numbers while preserving the network topology. The Z-score, see methods, of the enzymes that occur in eukaryotes is substantially higher than the Z-score for the enzymes which only occur in archaea and bacteria, see Figure 3b.

**Figure 4:**
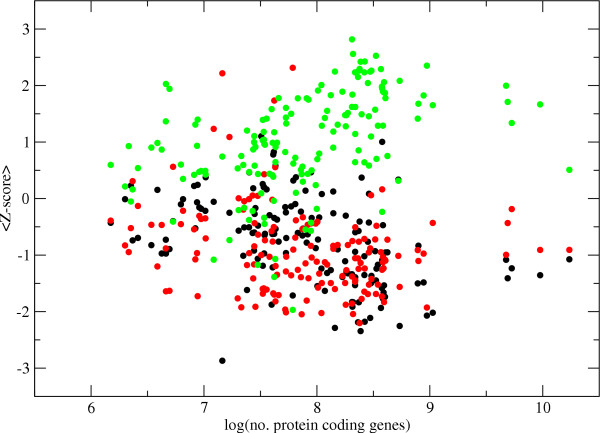
**Average Z-score and number of protein coding genes**. The average Z-score (between 15–20 compounds removed from the network) for enzymes which occur in 1 (black), 2 (red) and 3 (green) domains of life is plotted against the number of protein coding genes contained in the genome.

**Figure 5:**
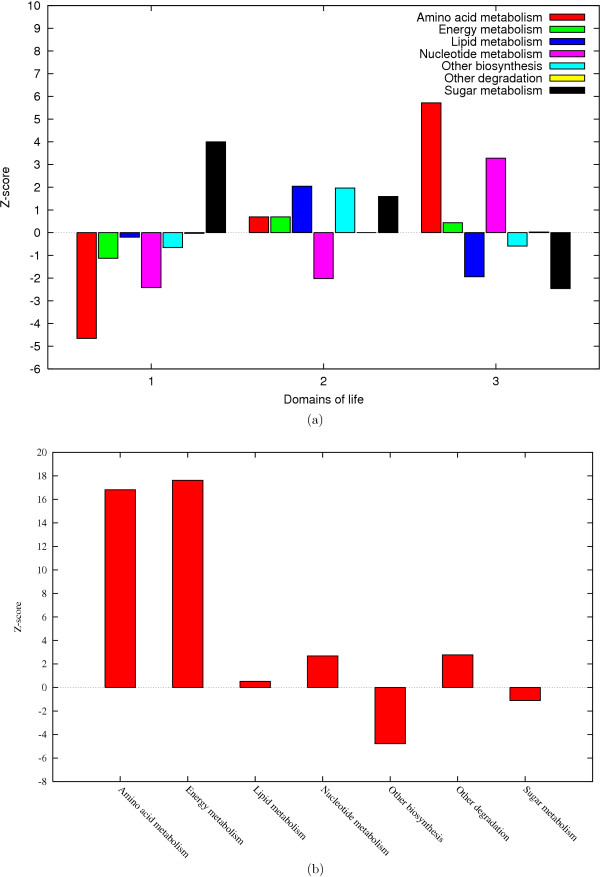
**The phylogenetic distribution and connectivity of enzymes in different functional classes**. a) Function and phylogenetic distribution. The *E. coli *enzymes were classified into 7 functional classes (amino acid metabolism, energy metabolism, lipid metabolism, nucleotide metabolism, sugar metabolism, other biosynthesis and other degradation) and divided into enzymes which are represented in 1, 2 or 3 domains of life. 100 000 randomized networks were generated for comparison and Z-score calculation. b) Connectivity and function. The enzymes were divided into functional classes and the Z-scores for the connectivities of each functional class were calculated for the network where the 15 most promiscuous compounds have been removed.

In a similar manner 147 other organisms were investigated. We collected the metabolic reactions for these organisms from KEGG. It should be noted that these metabolic networks have not been investigated to the extent of the *E. coli *metabolic network. Therefore, the connectivities of the enzymes and the results are probably not of the same reliability. We found that the larger prokaryotic genomes often have a particularly strong correlation between connectivity and domain presence while the correlation in smaller prokaryotes and eukaryotes is weaker, see Figure 4. Many of the smaller prokaryotes are obligate intracellular parasites or symbionts. The genomes of obligate parasites and symbionts have been metabolically reduced and many metabolic functions, such as the amino acid metabolism, are frequently provided by the host. Since enzymes involved in amino acid metabolism are often ancient proteins with high connectivities, see Figure 5a and 5b, the absence of genes coding for these enzymes in the genomes of the obligate symbionts/parasites can account for the lack of correlation between connectivity and domain presence in smaller genomes.

In conclusion we found that *E. coli *enzymes which have representatives in all domains of life, and in eukaryotes but not archaea, have a higher average connectivity in the metabolic network of *E. coli *than the presumably younger enzymes which only have representatives in γβ-proteobacteria. This finding lends support for one of the predictions of the mechanism of preferential attachment.

### Connectivity and horizontal gene transfer

It has been suggested that the scale-free properties of biological networks may arise, at least partially, as a result of preferential attachment of new nodes to highly connected nodes through gene duplication [13,14]. Preferential attachment by gene duplication may take place according to the following scenario; Initially, the duplicated gene has exactly the same function and position in the network as the template gene. Since many genes are connected to the hub of the network, the duplicated gene is by chance likely to be connected to the hub of the network. Subsequently, the duplicate gene may evolve towards another functionality but it could retain some of its original function. For instance, a multi-domain protein could loose one of its domains through deletion but retain the other domains and possibly part of its original functionality. In such a scenario the older proteins are more likely to be highly connected than the younger proteins.

An alternative scenario is preferential attachment by horizontal gene transfer (HGT); A new, or alternative, enzyme is introduced through HGT. The new enzyme is more likely to be retained in the metabolic repertory if it confers a new or improved function at a central, rather than peripheral, position of the metabolism – such as if it is connected to a highly connected enzyme or if it is itself highly connected. This is a consideration which may be particularly important in the metabolism of bacteria since some bacteria are prone to delete dispensable genes from their genomes [21]. Arguably, connectivity is a measure which indicates the centrality and importance of an enzyme in which case horizontally transferred genes should frequently be highly connected or be connected to highly connected enzymes. According to this scenario, horizontally transferred enzymes would be preferentially attached to highly connected enzymes and/or be preferentially replacing highly connected enzymes.

**Figure 6:**
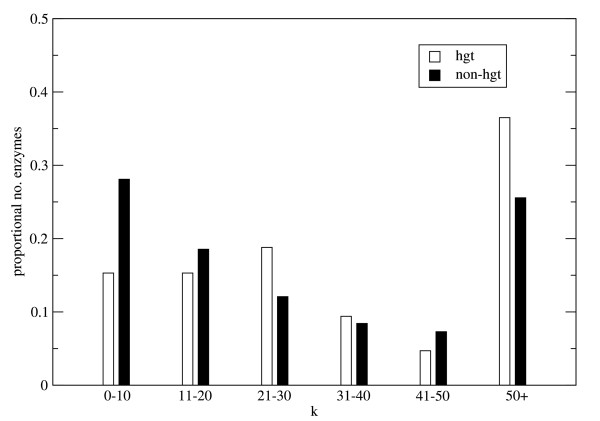
**Connectivity of HGT and non-HGT enzymes**. The proportion of the number of enzymes in each connectivity group is plotted against the binned connectivities for the enzymes which are candidates for horizontal gene transfer (white bars) and enzymes which are not candidates for HGT (black bars). The results are shown for the network where the 15 most promiscuous compounds had been removed.

Although the extent of the evolutionary impact of HGT is still under debate [22-24], it is generally accepted as an important evolutionary process in microbial species [19]. Roughly 18% of the protein coding genes in *E. coli *are likely to have been introduced into the *E. coli *genome by HGT since the species diverged from the *Salmonella *lineage according to an analysis by Lawrence and Ochman where base composition and codon usage patterns were used to identify the horizontally transferred sequences [25]. Using this data set we found that 85 of the 486 *E. coli *enzymes used in this study are likely to be examples of HGT (HGT enzymes). The average connectivity for the HGT enzymes in the metabolic network of *E. coli *is 42.2 while the other enzymes (non-HGT enzymes) have an average connectivity of 33.1. The most striking difference between the connectivity distributions of the HGT enzymes and the non-HGT enzymes is that only 15% of the HGT enzymes have connectivities between 0–9 and 37% of the HGT enzymes have higher connectivities than 60 while 28% of the non-HGT enzymes have connectivities of 0–9 and 26% have connectivities higher than 60, see Figure 6. These results indicate that horizontally transferred enzymes in the metabolic network of *E. coli *are either introduced into the repertory of the organism as a comparatively high connectivity enzyme or acquires many connections during the evolution of the network.

**Figure 7:**
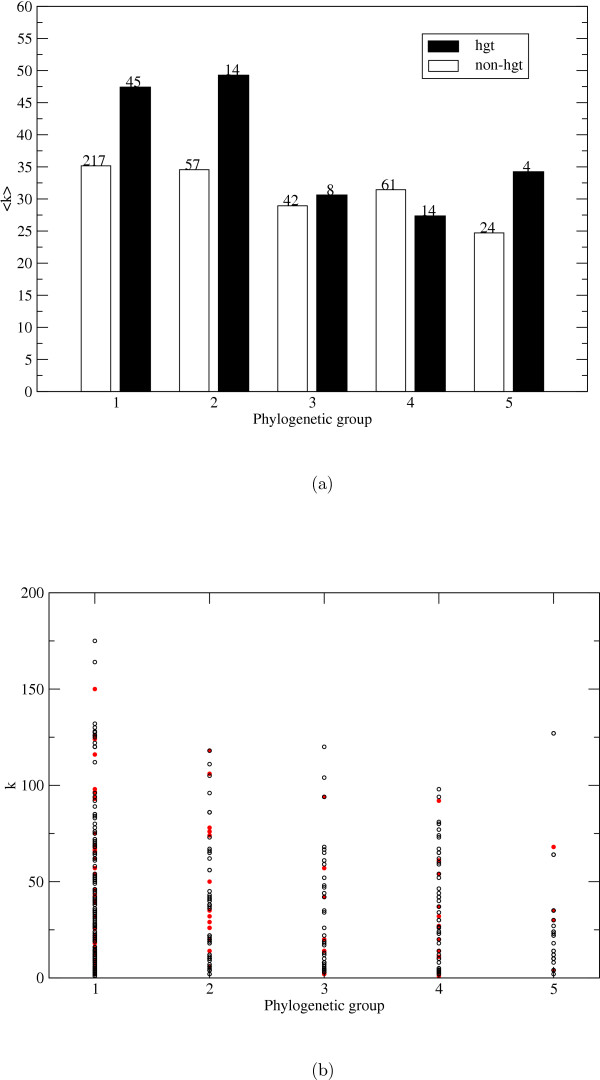
**Horizontal gene transfer, phylogenetic group and connectivity**. a) Average connectivities for enzymes in phylogenetic groups 1–5 in the metabolic network where the 15 most promiscuous compounds have been removed. The white bars represent the enzymes which have probably not been transferred to *E. coli *through horizontal gene transfer (HGT) and the black bars represent the enzymes which are likely to be examples of HGT. The numbers above the bars signify the number of enzymes in each group. b) The connectivity distribution for enzymes which are the result of horizontal transfer (red dots) and enzymes which are not (black circles).

We classified the HGT enzymes into the five phylogenetic groups and determined the average connectivities for each group, see Figure 7 and Table 1. Only HGT enzymes belonging to group 1 and 2 have higher connectivities than the non-HGT enzymes belonging to the same group. From Figure 7a it is clear that the high average connectivities of group 1 and 2 enzymes, which was seen in Figure 3a, is partly but not solely due to HGT enzymes. It should be noted that the horizontal transfers of enzymes which are specific for βγ-proteobacteria only (group 5) may be underestimated since HGT events between closely related species are hard to detect [24].

Most horizontally transferred genes go through the process of amelioration, the adjustment of the transferred sequence to the base composition and codon usage of the resident genome. Therefore, most detectable HGTs have taken place relatively recently in the history of *E. coli *[25]. Consequently, we can conclude that while it is true that the highly connected enzymes in the metabolic network of *E. coli *are often old in the sense that they are enzymes with representatives in eukaryotes, and which therefore probably originated in the last common ancestor of eukaryotes and bacteria, they are also overrepresented among the enzymes which have been introduced recently into the *E. coli *genome through HGT. These findings suggest that horizontally transferred genes are introduced and retained preferentially at central positions of the metabolism of *E. coli*.

### The connectivity of essential enzymes and isozymes

Jeong *et al *[26] showed that the highly connected proteins in the protein-protein interaction network of *S. cerevisiae *are more likely to be indispensable to the organism than less well connected proteins. We wished to study if there was a similar correlation in the metabolic network of *E. coli*. We used the essentiality classification from the study of Gerdes *et al *[27] of *E. coli *under aerobic growth in nutrition rich medium. We calculated the mean connectivity for the essential and the dispensable enzymes respectively and found that the essential enzymes do not show a higher connectivity than expected (, for networks where 15 compounds have been removed). It is possible that the relatively small size of the metabolic networks compared to the protein-protein interaction network is the reason a similar correlation could not be found in the metabolic network of *E. coli*.

The hubs are the most important nodes for the integrity of the network. If a fraction of the hubs are removed the network is likely to become fragmented into smaller components. Since these enzymes are very important for the robustness of the network it might be suspected that the EC numbers with the highest connectivities could have more than one representatives in the genome, i.e. that there are two or more isozymes representing these highly connected nodes. Isozymes in multicellular organisms are often active in different tissues while isozymes in single cellular organisms frequently have different substrate specificities or are activated in different environments (such as aerobic or anaerobic environments). We here designated a pair of enzymes as isozymes if they catalyze the same reaction but are coded for by different genes, which are not part of the same enzyme complex.

We used Expasy [28], SGD [29] and EcoCyc [17] to determine which enzymes in the metabolic networks of *E. coli *and *S. cerevisiae *occur as isozymes. We found 77 EC numbers that were associated with isozymes in *E. coli *and 97 EC numbers that were associated with isozymes in *S. cerevisiae*, see additional files. The mean connectivities for the isozymes and the non-isozymes were determined and the result was compared to randomized networks. We found that the isozymes do not have a noticeably higher mean connectivity than non-isozymes (, for networks where 15 compounds have been removed). The result may indicate that isozymes are not necessarily crucial for the integrity of the metabolic network. In accordance with our result it has recently been shown that the isozymes of *S. cerevisiae *are not overrepresented among essential enzymes [30].

### Connectivity and function

Kunin *et al *showed that the functional classes in the protein-protein network of *S. cere-visiae *display distinctly different connectivity levels [16]. In a similar manner we investigated whether enzymes belonging to different functional groups are characterized by distinct connectivities.

We classified the enzymes into 7 functional classes according to EcoCyc [17]; lipid metabolism, nucleotide metabolism, amino acid metabolism, sugar metabolism, energy metabolism, other biosynthesis and other degradation and calculated the mean connectivities for the different functional classes, see Figure 5b and Table 2. The mean connectivities for the enzymes involved in nucleotide, amino acid, other degradation and energy metabolism are higher than expected. The amino acid metabolism and nucleotide metabolism enzymes are clearly over represented in 3 domains of life while enzymes involved in energy metabolism are slightly more common in 3 domains of life than expected by chance. Many of the pathways involved in energy metabolism, such as the citric acid cycle and glycolysis, are believed to be very old. However, there are substantial variations in the energy metabolism between different species and domains of life [31]. Therefore, the observation that energy metabolism enzymes are not overrepresented in three domains of life is not surprising.

**Table 2:** The number of *E. coli *enzymes belonging to 7 functional EcoCyc classes.

FUNCTION	NO. ENZYMES
Sugar metabolism	76
Amino acid metabolism	93
Lipid metabolism	17
Nucleotide metabolism	46
Energy metabolism	46
Other biosynthesis	140
Other degradation	53

Contrastingly, enzymes involved in lipid and sugar metabolism are on average half as well connected as the enzymes involved in nucleotide, amino acid and energy metabolism. The group of enzymes involved in lipid metabolism is less than half the size of the second smallest functional group and due to its small size the Z-score for this functional group is less reliable than for the other functional groups. The sugar metabolism enzymes are clearly over represented among the enzymes that occur in bacteria only, see Figure 5a, which was anticipated since there are many bacterial specific enzymes involved in sugar transportation [32].

### Network growth through preferential attachment

**Figure 8:**
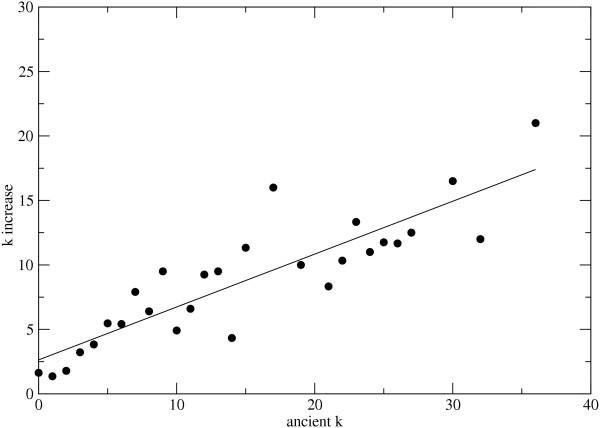
**Connectivity increase and connectivity in the ancient network**. The number of edges gained from the ancient network to the current *E. coli *metabolic network is plotted against the connectivity in the ancient network.

According to the mechanism of preferential attachment new enzymes in the network should be preferentially attached to already well connected nodes. We do not have access to the last universal common ancestor (LUCA) that existed before the 3 domains of life evolved. However, a rough representation of the metabolic network of that organism was created by extracting the enzymes that occur in all domains of life. The connectivities of the enzymes in that derived network were determined. We then calculated the number of enzyme nodes that have been added to an enzyme by subtracting the connectivity of the enzyme in the current *E. coli *network by the connectivity of the enzyme in the ancient network. We found that the enzymes which have higher connectivities in the ancient network gain new connections at a higher rate than the enzymes with lower connectivities, see Figure 8. The correlation between connectivity in the ancient network and the connectivity increase appears to be linear (r = 0.87) following the equation *f*(*x*) = 2.6 + 0.41*x*, where x is the connectivity in the ancient network. We can therefore conclude that the addition of new nodes to the metabolic network of *E. coli *occurs in a manner which is consistent with preferential attachment.

## Discussion

We have investigated two predictions generated from the mechanism of preferential attachment in the evolution of the metabolic network of *E. coli*. First, if preferential attachment is of any significance in the evolution of the metabolic network of *E. coli*, the older enzymes in the network should have a higher average connectivity. We have found that *E. coli *enzymes which are represented in three domains of life, and in eukaryotes but not archaea, have a higher average connectivity than expected by chance. Second, another prediction generated from the hypothesis of network evolution through preferential attachment is that highly connected nodes should gain new edges at a faster rate than nodes with low connectivities. To investigate this prediction we extracted the enzymes with representatives in 3 domains of life and determined the network representing LUCA's metabolic network. In accordance with the mechanism of preferential attachment we found a positive linear correlation between connectivity in the ancient network and number of connections gained through evolution.

Further, we found that the *E. coli *enzymes which are believed to have undergone horizontal gene transfer (HGT enzymes) have a higher average connectivity than other enzymes (non-HGT enzymes). This is especially true for the HGT enzymes with representatives in eukaryotes, which is the most highly connected group of *E. coli *enzymes. This result suggests that the highly connected enzymes are often old in the sense that they are likely to have originated in LUCA and been part of the bacterial metabolic repertory for a long time. However, these ancient enzymes are sometimes relatively recent additions to the metabolic network of *E. coli*. It is possible that bacteria such as *E. coli *are adjusting their metabolic networks to a changing environment by replacing the relatively central enzymes, with high connectivities, for better adapted orthologs from other prokaryotic species.

## Conclusion

It is well known that many novel functions in organisms are obtained through gene duplication, followed by subfunctionalization and neofunctionalization. Therefore, a possible biological explanation for the preferential attachment growth of metabolic networks, which we have now found some support for, could be that novel enzymes, which are created through gene duplication, maintain some compounds involved in the reaction catalyzed by the original enzyme throughout its future evolution. As a supplementary explanation we propose that horizontally transferred enzymes are introduced preferentially at central positions of the metabolic network of *E. coli*.

## Methods

### Databases and representation framework

We built a representation of the metabolic network of *E. coli *by using EcoCyc [17] (downloaded in March 2004) to gather the EC assigned enzymes and to determine the connectivities of the enzymes. An alternative network based on KEGG was also produced and the study was performed which generated similar results, results not shown. The connectivity of an enzyme is defined as the number of edges connecting the enzyme to other enzymes. Only one edge in each direction between any two enzymes was allowed. Furthermore we used KEGG orthology (KO) assignments [18] (downloaded in May 2004) to determine in which organisms the different EC numbers are represented.

The nodes in our graph represent the enzymes (complete EC numbers) catalyzing the reactions and the edges represent one or more compounds involved in the reactions. There is an edge from enzyme El to enzyme E2 if El catalyzes a reaction where compound A is produced and then E2 uses A as substrate. The network representation used in our study has been used before for metabolic network analysis where it has been referred to as 'protein-centric' graphs [33] or 'reaction graphs' [7], see Figure 1b. Our representation of the full metabolic network of *E. coli *consists of 486 nodes and 99 917 edges.

One problematic aspect with metabolic network analysis is how promiscuous compounds, such as H_2_O, should be handled. One may argue that the network would become more biochemically meaningful if these compounds are removed because the promiscuous compounds are usually not limiting factors of reactions [34]. In this study, we have chosen to apply a simple network-based criterion. We count the number of times a compound occurs as part of an edge in the network. The most common compounds were then considered as promiscuous compounds [2,3]. We performed our studies on different networks where up to 40 compounds have been removed.

### Statistical analysis

For the statistical analysis 100 000 randomized networks were generated through shuffling the group numbers while preserving the network topology. Subsequently, Z-scores were calculated. The Z-score expresses how far the average connectivity of the enzymes belonging to a certain phylogenetic group differs from the average connectivity of randomly sampled enzymes, measured in units of the random sampling distribution's standard deviation. The larger the Z-score, the less likely that the difference between phylogenetic group's average and the random group's average is by chance.

For the calculations of the Z-score for the average connectivity for phylogenetic groups the Z-score is defined as , where i is the phylogenetic group and  is the average connectivity.

For the calculations of the Z-score for the average connectivity for functional groups the Z-score is defined as , where f is the functional group and  is the avera ge connectivity.

For the calculations of the Z-score for the number of members belonging to functional groups per domain of life the Z-score is defined as , where d is the number of domains of life and  is the number of enzymes belonging to each functional class.

## Authors' contributions

SL performed the analysis as a graduate student under the supervision of PK and AE.

## Supplementary Material

Additional File 1 ***E. coli *isozymes **Flat file containing a simple list of the isozymes in *E. coli*. Click here for file

Additional File 2 ***S. cerevisiae *isozymes **Flat file containing a simple list of the isozymes in *S. cerevisiae*. Click here for file
